# Intracorporeal renal hypothermia with ice slush for robot-assisted partial nephrectomy in a highly complex renal mass

**DOI:** 10.1590/S1677-5538.IBJU.2018.0705

**Published:** 2019-01-29

**Authors:** Jose Luis Bauza, Prithvi Murthy, Daniel Sagalovich, Riccardo Bertolo, Enrique Pieras, Pedro Piza, Jihad Kaouk

**Affiliations:** 1 Department of Urology Hospital Universitario Son Espases Palma de Mallorca Illes Balears Spain Department of Urology, Hospital Universitario Son Espases, Palma de Mallorca, Illes Balears, Spain;; 2 Center for Laparoscopic and Robotic Surgery Glickman Urological & Kidney Institute Cleveland Clinic Cleveland Ohio United States Center for Laparoscopic and Robotic Surgery, Glickman Urological & Kidney Institute, Cleveland Clinic, Cleveland, Ohio - United States

## CASE DESCRIPTION

### Objective

To report our step-by-step technique for robotic partial nephrectomy using intracorporeal renal hypothermia (RPNIRH) in a highly complex renal mass. The robotic technology has allowed surgeons to recreate the principles of open surgery in a minimally invasive approach (1). With increasing experience, larger deeply infiltrative tumors can be treated with this technique (2). In complex cases, when a long warm ischemia time is expected, intracorporeal renal hypothermia can be useful to prevent permanent renal function loss (3).

## MATERIALS AND METHODS

A 69 years old male with chronic kidney disease with an atrophic left kidney, appendectomy and a right ureteral reimplant due to an ureteral stenosis was incidentally found a right renal mass, 8.5 cm in diameter, cT2a, RENAL score 11p and several retroperitoneal lymph nodes <1cm. Neoadjuvant therapy with tyrosine kinase inhibitors and subsequent partial nephrectomy was recommended by the Urologic-Oncology tumor board. No tumor shrinkage was evident in the control imaging. Thus the patient underwent a RPNIRH.

## RESULTS

Operative time was 185 min. Cold ischemia time was 49:50 min. Average kidney temperature was 24.3ºC. Blood losses were negligible and no postoperative complications appeared, eGFR at discharge was 14ml/min/1.73m^2^. Final pathology revealed a clear cell renal cell carcinoma, pT3aN0, ISUP grade 3, involving the sinus fat. Surgical margins were negative.

## CONCLUSIONS

The RPNIRH using ice slush is simple, highly reproducible and may improve postoperative renal function in the short term. Consistent experience is needed before embarking on this surgery. Reports on long-term oncological outcomes for such lesions are awaited.
